# Cold spells in the Nordic Seas during the early Eocene Greenhouse

**DOI:** 10.1038/s41467-020-18558-7

**Published:** 2020-09-18

**Authors:** Madeleine L. Vickers, Sabine K. Lengger, Stefano M. Bernasconi, Nicolas Thibault, Bo Pagh Schultz, Alvaro Fernandez, Clemens V. Ullmann, Paul McCormack, Christian J. Bjerrum, Jan Audun Rasmussen, Iben Winther Hougård, Christoph Korte

**Affiliations:** 1 IGN, University of Copenhagen, Øster Voldgade 10, DK-1350 Copenhagen, Denmark; 2 Biogeochemistry Research Centre, School of Geography, Earth and Environmental Sciences, Plymouth University, Drake Circus, Plymouth, PL4 8AA UK; 3 ETH Zurich, Geologisches Institut, Sonneggstrasse 5, 8092 Zürich, Switzerland; 4 Museum Salling, Fur Museum, Nederby 28, 7884 Fur, Denmark; 5 Bjerknes Centre for Climate Research and Department of Earth Science, University of Bergen, Allégaten 41, N-5007 Bergen, Norway; 6 Camborne School of Mines, University of Exeter, Penryn Campus, Penryn, Cornwall, TR10 9FE UK; 7 Museum Mors, Fossil- and Mo-clay Museum, Skarrehagevej 8, 7900 Nykøbing Mors, Denmark

**Keywords:** Palaeoceanography, Palaeoclimate, Environmental chemistry

## Abstract

The early Eocene (c. 56 - 48 million years ago) experienced some of the highest global temperatures in Earth’s history since the Mesozoic, with no polar ice. Reports of contradictory ice-rafted erratics and cold water glendonites in the higher latitudes have been largely dismissed due to ambiguity of the significance of these purported cold-climate indicators. Here we apply clumped isotope paleothermometry to a traditionally qualitative abiotic proxy, glendonite calcite, to generate quantitative temperature estimates for northern mid-latitude bottom waters. Our data show that the glendonites of the Danish Basin formed in waters below 5 °C, at water depths of <300 m. Such near-freezing temperatures have not previously been reconstructed from proxy data for anywhere on the early Eocene Earth, and these data therefore suggest that regionalised cool episodes punctuated the background warmth of the early Eocene, likely linked to eruptive phases of the North Atlantic Igneous Province.

## Abstract (web summary)

The early Eocene was characterized by exceptionally high global temperatures and no polar ice. Here, clumped isotope paleothermometry of glendonite calcite from the Danish Basin shows that these were formed in waters below 5 °C, indicating that regionalised cool episodes punctuated the background warmth of the early Eocene.

## Introduction

The Eocene, 56.0 – 33.9 Ma, is the final epoch with global greenhouse conditions that dominated the Mesozoic and earliest Cenozoic^1,2^. Despite a large body of evidence for globally warm background conditions throughout the early Eocene^2,3^, there are reports of glacial sediments and glendonites from the high latitudes, suggesting that episodic cooler intervals may have interrupted the Eocene warmth^4–6^.

“Glendonite” is the name given to the calcite pseudomorphs after the mineral ikaite (CaCO_3_·6H_2_O), a hydrated form of calcium carbonate that in general is unstable at Earth surface temperature and pressure conditions^7^. A combination of low temperatures (≤4 °C in the natural marine sedimentary realm^8^), and chemical inhibitors of the more stable polymorphs, are needed to promote the precipitation and stabilisation of ikaite over calcite and aragonite^9^. In the marine sedimentary realm, ikaite growth is thought to be facilitated by either the anaerobic oxidation of methane (AOM) or bacterial sulphate reduction, both of which generate high alkalinity and high phosphate and/or sulphate concentrations which may act as chemical inhibitors of the anhydrous CaCO_3_ polymorphs^8,10,11^. The carbon source (AOM or organic matter) is reflected in the δ^13^C signatures of the ikaite-derived glendonite calcite^10,12^.

Glendonites are traditionally considered cold-climate indicators, due to the temperature-dependency of their parent ikaite in natural settings, and the fact that they are found in paleo-high latitude sediments frequently associated with glacial deposits^13^. Their presence in early- and mid-Eocene deposits from North America^14^ (paleolatitude c. 55 °N^15^); the Arctic^4^ (paleolatitude c. 77 °N^15^); and Denmark^5,16^ (paleolatitude c. 45 °N^15^) is something of a quandary. The glendonites and potentially ice-rafted erratics found in the Paleocene-Eocene strata of Svalbard have given rise to speculation of multiple transitions between cold and warm phases during this time^4^. However, since the successful laboratory growth and stabilisation of synthetic ikaite at 35 °C^9^, the significance of glendonites in the geological record has been questioned. No truly quantitative temperature reconstructions were possible for the Svalbard glendonites due to the poor preservation of the entire succession on Svalbard^4^.

The Fur Formation diatomite of the early Eocene of Denmark is renowned for its exceptionally well-preserved marine and terrestrial fossils^5^. The flora and fauna of the Fur Formation suggest local tropical to sub-tropical climates^5^. Thus, the presence of numerous, unusually large glendonites at several levels in the succession has long been a conundrum^16^, and appears to support the findings that ikaite may grow at much higher temperatures than near-freezing^9^. Quantitative temperature reconstructions for the glendonites are needed to test if the glendonites of early Eocene Denmark are indeed indicative of very low temperatures (≤4 °C^8^).

## Geological setting

**Fig. 1:**
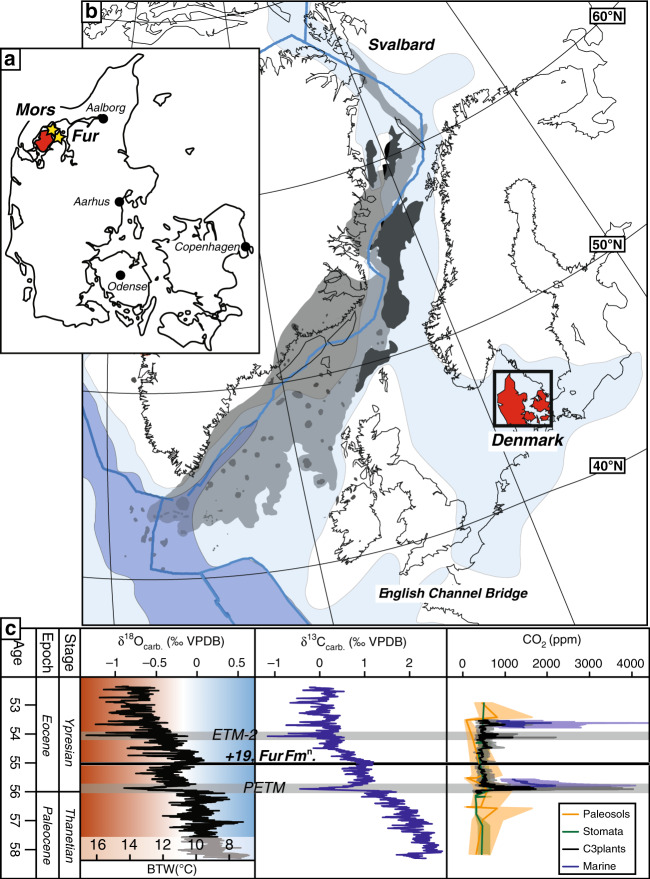
Summary of relevant geographical and geochemical data. **a** current geographic map of the study area. **b** Paleogeographic reconstruction of the North Sea Basin and surrounding regions, made using the paleomagentic reference frame^15^; NAIP and other details from Jones et al.^18^. **c** Position of Fur Formation (+19 horizon), in relation to the Eocene hyperthermals^53^, stable C- and O- isotope records^55^, and reconstructed *p*CO_2_^54^. Geological timescale of Ogg et al.^85^.

**Fig. 2:**
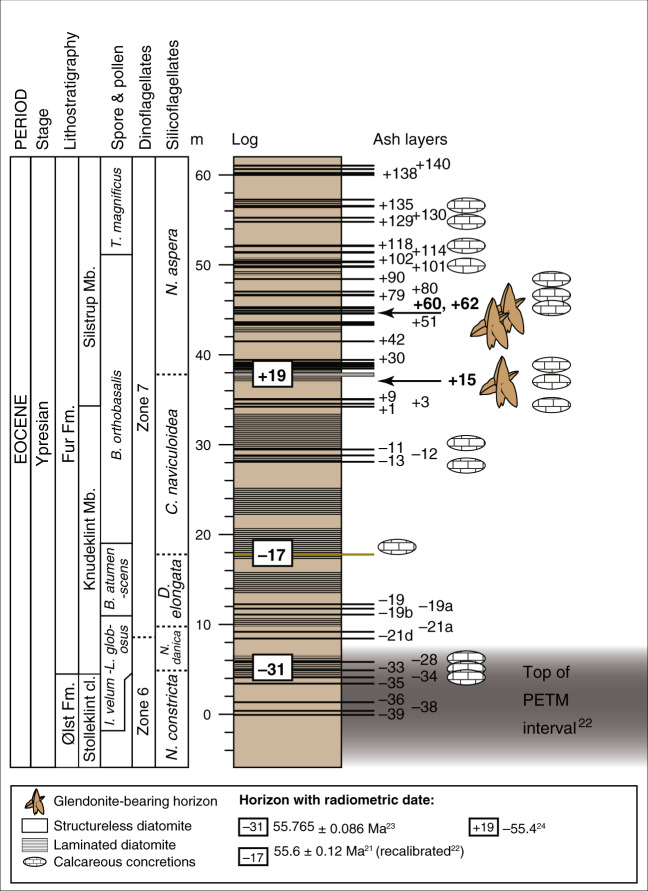
Idealised graphic log for the Fur Formation. The numbers (e.g. +15, −39) refer to the ash layers. The PETM interval lies 24 m below ash layer −33. Dated ashes from Storey et al.^21^, Westerhold et al.^24^, and Charles et al.^23^. Spore and pollen zones from Willumsen^77^, dinoflagellate cyst zones from Heilmann-Clausen et al.^86^ and silicoflagellate zones from Perch-Nielsen^87^. Fm. Formation.

The approx. 60 m thick interbedded diatomite and ash horizons of the Fur Formation are exposed in northern Denmark, c. 56°56′ N; 8°54′E (Fig. 1a), in glacially folded and thrusted outcrops^17^. The formation is subdivided into the laminated Knudeklint Member and overlying Silstrup Member, which has a higher proportion of structureless diatomite^17^ (Fig. 2). The 179 numbered volcanic ash layers were deposited during the emplacement of the North Atlantic Igneous Province^18,19^ (NAIP; Fig. 1b), and represent isochronous horizons^20^. The glendonites analysed here (Supplementary Figs. 1 and 2) are found in ash layers +15, +60 – +62^16^, which are thought to have been deposited between the Paleocene-Eocene thermal maximum (PETM) and Eocene thermal maximum 2 (ETM2), ~55 Ma (Fig. 1c). The age constraints for the Fur Formation come from several dated NAIP ash layers in the region (Fig. 2). Ash layer -17 was dated in 2007 by Ar/Ar as 55.1 Ma^21^. Since then, the calibration for the Fish Canyon Tuff has changed and the recalibrated age is closer to 55.6 ± 1 Ma Stokke et al.^22^ . An ash layer in Svalbard, equivalent to the -31 layer in the Fur Formation^18^ has been dated at 55.765 ± 0.086 Ma^23^. Closer to the glendonite-bearing horizons, Westerhold et al.^24^ place ash horizon +19 at about 55.4 Ma, based on cyclostratigraphy. The position of the glendonite-bearing-horizons relative to these dated ash layers are shown on Fig. 2.

During the Eocene, the semi-enclosed Danish Basin was open to the Greenland Sea in the northwest, with possible, shallow openings to the east and southwest^15,18,25^ (Fig. 1b). The main influx of marine water to the Danish Basin was likely only from the north, despite uncertainties about whether it was partially open to the east^18,26^. Where this influxing water was ultimately derived from, or if the Nordic seas region was even entirely enclosed, remains unclear^15,18,27,28^ (Fig. 1b). The current model for the Fur Formation depositional environment was developed by Bonde^29^, based on a number of lines of evidence pointing to upwelling occurring in the basin. Pedersen^30^ suggested a more locally restricted upwelling model than that of Bonde^29^.

In this study, we apply clumped isotope thermometry to hand-separated ikaite-derived glendonite calcite, to show that temperatures in the Danish Basin were near-freezing during particular intervals of the early Eocene. These results, in conjunction with other geochemical data from the Fur Formation and by comparison to published marine and terrestrial temperatures during this time, indicate at least regionalised cooling to near-freezing temperatures in the Nordic Seas region during the early Eocene Greenhouse, possibly linked to NAIP volcanism.

## Results

### Glendonite characterisation

**Fig. 3:**
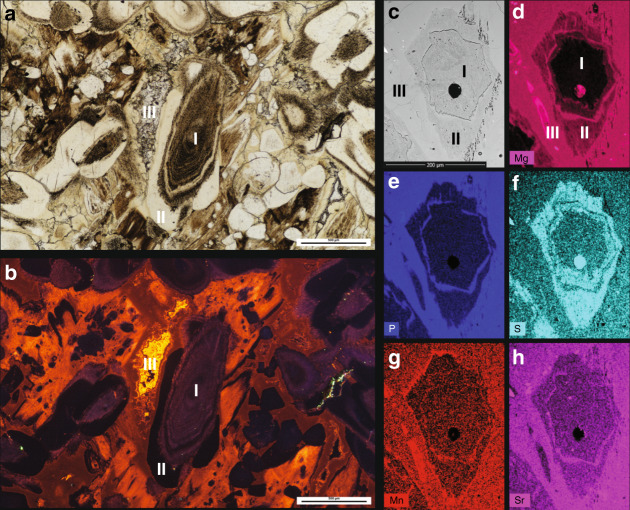
Glendonite thin-section photomicrographs. **a** Thin section of a glendonite from the +60–62 horizon under plane polarised light. **b** The same view under cathodoluminescence. The three main calcite phases (types I, II and III, as defined in Vickers et al.^12^) are clearly distinct, showing different luminescent properties. **c** Secondary Electron SEM image of thin section of glendonite from the +15 horizon. **d**–**h** Element maps (by Energy Dispersive X-Ray Spectroscopy, EDS) of the same view, showing Mg, Mn, P, S and Sr variations. I = Type I calcite, the ikaite-derived calcite phase in glendonite; II = secondary calcites, collectively called Type II calcite, believed to have grown rapidly on the Type I calcite after transformation of the ikaite to calcite; III = Type III calcite, a late-phase spar infilling pore spaces in glendonite.

**Fig. 4:**
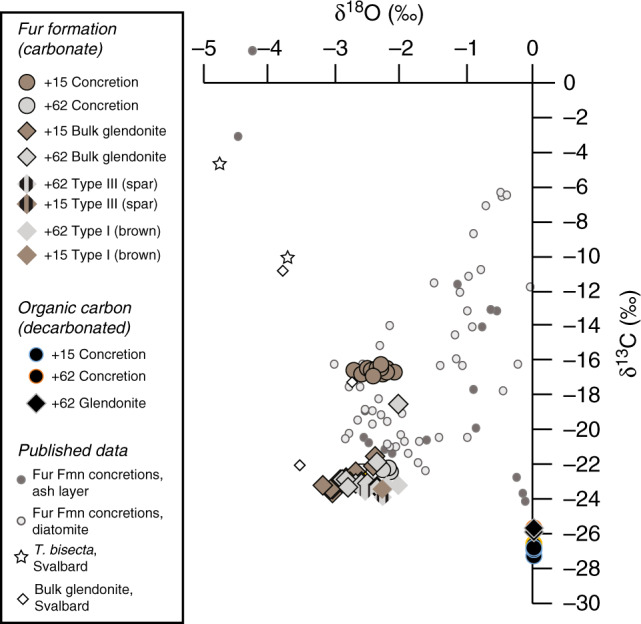
Glendonite and concretion stable isotope data. Plotted stable isotope data for +15 and +60–62 horizons in the Eocene Fur Formation glendonite and concretions, along with δ^13^C data for organic matter from decarbonated glendonites and concretions. For comparison, published stable isotope data for concretions of the Fur Formation and glendonites from the early Eocene succession on Svalbard (Basilika Fmn) are also shown^4,5^.

**Fig. 5:**
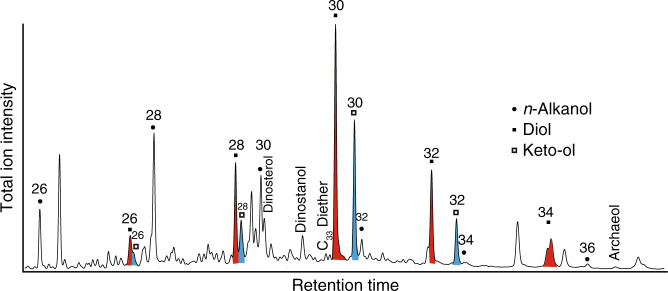
Biomarker distribution for +15 concretion. Chromatogram showing the biomarker distribution from +15 concretion.

The characterisation of the glendonites is based on Scanning Electron Microscopy (SEM), cathodoluminescence (CL) and light microscopy (Fig. 3), stable isotope (Fig. 4) and minor element analysis (Table 1), clumped isotope thermometry (Table 1), and organic biomarker extraction (Table 2 and Fig. 5), with details of the analyses in the Methods section.

**Table 1:** Summary of inorganic geochemical data.

Analyses performed at:			ETH, Zurich							University of Copenhagen			CSM, Exeter University						
Calcite type	Ash horizon	Replic-ates	Δ_47_	Δ_47_ SD	Temp. °C	Temp. °C 95% CL	δ^13^C_c_ VPDB	δ^18^O_c_ VPDB	δ^18^O fluid VSMOW	δ^13^C_org_ VPDB	δ^13^C_c_ VPDB	δ^18^O_c_ VPDB	Ca wt%	Mg/Ca mmol/mol	Sr/Ca mmol/mol	Mn/Ca mmol/mol	Fe/Ca mmol/mol	S/Ca mmol/mol	P/Ca mmol/mol
Glendonite calc. Type I	+15	14	0.766	0.036	0.9	4.7	−23.38	−2.27	−5.17										
Glendonite calc. Type III	+15	11	0.734	0.031	8.6	5.1	−23.12	−2.70	−3.44										
Bulk glendonite	+15	Not measured as gives mixed signal of the different calcite types									−22.90	−2.79	36.2	28.3	1.14	3.6	1.4	1.6	5.5
Bulk concretion	+15	15	0.744	0.020	5.8	2.7	−18.53	−2.02	−3.77	−26.95	−17.15	−2.35	31.9	37.4	1.35	10.6	17.1	6.1	12.5
Empty concretion	+15	10	0.756	0.031	3.2	5.1	−18.27	−2.02	−4.39	−26.59									
Glendonite calc. Type I	+60–62	16	0.732	0.027	9.1	3.7	−23.23	−2.19	−3.21										
Glendonite calc. Type III	+60–62	14	0.714	0.026	13.6	4.0	−23.51	−2.52	−2.52										
Bulk glendonite	+60–62	Not measured as gives mixed signal of the different calcite types								−25.76	−22.43	−2.55	35.9	30.0	1.32	3.8	3.6	5.8	10.6
Bulk concretion	+60–62	14	0.729	0.049	10.3	8.3	−21.97	−1.96	−2.69	−25.60	−22.55	−2.52	33.3	33.6	1.35	3.8	9.8	10.4	9.9
Bulk concretion	+60–62	Not measured																	

Averaged results for clumped and stable isotope and Element/Ca ratio analyses of glendonites, concretions and sediment.

*SD* standard deviation, *Temp.* Temperature, *CI* Confidence Interval, *VPDB* Vienna PeeDee Belemnite, *VSMOW* Vienna Standard Mean Ocean Water.

**Table 2:** Mean averaged biomarker data.

Sample type	Ash horizon	Long chain diols				Alkenones	GDGT SST			Quality control					
		LDI	Diol index 2	%C_32_	%C_30_	C_37_/C_38_	TEX_86_	BAYSPAR	TEX_86_^H^	BIT	GDGT-2/cren	MI	RI	RI_TEX_	ΔRI
bulk															
Concretion A	+15	0.84	0.61	24.63	55.88	3.98	0.476	14.09	16.52	0.08	0.06	0.16	1.87	1.97	0.10
Concretion B	+15	0.84	0.61	24.89	55.60	4.05	0.476	14.09	16.56	0.07	0.05	0.13	1.97	1.98	0.01
Sediment	+15	0.84	0.62	24.89	55.66	3.04				0.70	0.09	0.18	2.51	2.03	0.48
Glendonite	+15	0.85	0.70	23.82	54.67	3.72	0.468	13.50	16.05	0.10	0.05	0.13	1.82	1.96	0.13
Concretion A	+60–62	0.78	0.35	34.78	56.56	3.08	0.437	11.37	14.04	0.20	0.05	0.14	1.99	1.89	0.10
Concretion B	+60–62	0.74	0.53	32.30	46.28	3.10	0.416	9.60	12.56	0.22	0.04	0.13	2.00	1.84	0.16
Sediment	+60–62	0.67	0.39	26.68	55.52	2.10				0.58	0.09	0.22	2.30	2.30	0.01
decarb. bulk															
Concretion A	+15	0.84	0.61	24.63	55.88	3.93	0.491	15.00	17.47	0.08	0.06	0.15	1.91	2.01	0.10
Concretion B	+15	0.84	0.61	24.89	55.60	3.95	0.481	14.40	16.83	0.07	0.05	0.13	1.91	1.99	0.07
Sediment	+15	0.84	0.62	24.89	55.66	3.11				0.61	0.09	0.18	2.55	2.12	0.43
Glendonite	+15	0.85	0.70	23.82	54.67	3.69	0.476	14.09	16.55	0.08	0.04	0.11	1.87	1.98	0.10
Concretion A	+60–62	0.78	0.35	34.78	56.56	2.98	0.455	12.63	15.24	0.20	0.05	0.13	2.02	1.93	0.09
Concretion B	+60–62	0.74	0.53	32.30	46.28	3.07	0.407	8.00	11.92	0.19	0.04	0.13	1.97	1.83	0.14
Sediment	+60–62	0.67	0.39	26.68	55.52	2.17				0.58	0.09	0.20	2.38	2.28	0.10

Full results in the Supplementary dataset.

*LDI* Long-chain Diol Index, *SST* Sea Surface Temperature (calculated from LDI^36^; TEX_86_^31^), *Diol Index 2* a proxy for upwelling intensity at high latitudes^74^, *FC*_*32*_Fractional abundance of C_32_ 1,15-diol, a proxy for riverine input^38^. *BIT* branched and isoprenoid tetraether index, and proxy for terrigenous input^32^, *ΔRI* the sample’s offset from the modern TEX_86_-Ring Index (RI) core top relationship^33^; *MI* Methane Index^43^.

### Clumped isotope thermometry

The ∆_47_ values for the Fur Formation carbonates range from 0.71 (±0.03) ‰ to 0.77 (±0.04) ‰ (Table 1). The ∆_47_ values yield average temperatures of 0.9 °C (±4.7 at the 95% confidence level) and 9.1 °C (±3.7) for the Type I (ikaite-derived) calcite of +15 and +60–62 glendonites, respectively. The Type III (late phase sparry) calcite yielded temperatures of 8.5 (±5.1) °C for +15 glendonite and 13.6 (±4.0) °C for the +62 glendonite. The calcite concretions yielded mean temperatures of 4.5 (±3.9) °C and 10.3 (±8.3) °C for +15 and +60–62 horizons, respectively.

### Biomarkers

Biomarker distributions for the sediment, concretion and glendonite samples are shown in Fig. 5 and Supplementary Figs. 6 and 7. Sea surface temperatures (SSTs) were reconstructed using GDGTs (TEX_86_ using the BAYSPAR calibration^31^). In the +15 horizon, temperatures reconstructed from the glendonites and concretions ranged from 13 to 15 °C; for the +60–62 horizon, the range was 8–12 °C. However, both sediment samples showed a BIT index above 0.4^32^ and ΔRI > 0.3^33^ (which identify samples with high amounts of terrigenous GDGTs or anomalous distributions, respectively), and were thus excluded from further interpretation (Table 2 and Supplementary Information dataset). This anomalous distribution or high terrestrial GDGT content may be due to the sediment samples representing a greater time period than the concretions within the horizon: the soft sediment experienced more compaction than the cemented concretions; and during this additional time significant terrestrial input to the sediment could have occurred. The concretions preserved only the planktonic-derived GDGTs in the sediment at the time of concretion formation^34,35^. Attempts of SST reconstruction using the Long Chain Diol Index (LDI^36^ and U^K’^_37_^37^, both gave high values; however, these proxies are unreliable as they are calibrated for open marine settings^38^. Additionally, while diols have been detected in Eocene samples^39^, their distribution is markedly different from the modern suggesting a different producer regime, and LDI has not been applied in this time period for this reason. Our biomarker data show high C_37_/C_38_ alkenone ratios (≥1.8, characteristic of coastal/brackish Group II haptophyte production^40^), and relatively high %C_32_1,15 diol (> 10 %, along with fairly low %C_30_1,15 diol), which is consistent with a strong riverine-influence^41^. Along with published floral and faunal data^5^, these data indicate a coastal, freshwater-influenced/brackish depositional environment, and therefore these open-ocean calibrated proxies (LDI and U^K’^_37_) cannot be reliably used for SST reconstructions in this study^38,40^.

## Discussion

The glendonites contain higher P and Mg concentrations than the concretions in which they are found, suggesting that these elements were inhibiting calcite and allowing ikaite nucleation in sediment^8,9^ (Fig. 3). Along with high Fe concentrations, the δ^13^C signature of the glendonites suggest that either c. 90% of the glendonite carbon was sourced from organic matter carbon, with 10% from dissolved inorganic carbon (DIC), or c. 50% was from methane and 50% from DIC (Fig. 4). These data thus imply that high amounts of bacterial sulphate reduction, rather than methane seep activity, generated the conditions favourable for ikaite growth^8,10,11^. This conclusion is supported by the biomarker distributions of extracted bulk undecarbonated glendonite, concretions and sediment. The presence, yet low abundances, of archaeol and diether lipids (Fig. 5) may be indicative of some diffusive methane flux^41^, but as photosynthetically-derived biomarkers dominate the signal, methane fluxes are thought to have been substantially lower than in a cold seep^42^. Furthermore, the Methane Index is very low (≤0.2), suggesting no AOM^43^. This is consistent with the GDGT data of Stokke et al.^22^.

The δ^18^O combined with the clumped isotope data allows reconstruction of the δ^18^O of the fluid (δ^18^O_w_) from which the calcite precipitated. All calcites give reconstructed δ^18^O_w_ lower than −1‰ (the average composition of seawater for an ice-free world^44^ (Table 1). Yet as the parent ikaite grew in the pore waters below the sea floor, it is more likely that this δ^18^O_w_ reflects early diagenetic conditions. The original bottom water δ^18^O signal could have been shifted to even lower δ^18^O_w_ by freshwater influence, as indicated by biomarkers and floral data. An additional potential cause for a negative shift of the δ^18^O is early diagenetic alteration of the abundant volcanic detritus^45^.

The new clumped isotope temperatures recording the crystallisation temperature of the calcite from ikaite have a mean value of 5 ± 4 °C, and are within error of the carbonate concretions (6 ± 5 °C). This fits well with what we know about ikaite formation and transformation to glendonite calcite. Given the uncertainty on these temperatures, these data are consistent with observations of modern marine sedimentary ikaite being unstable above 4 °C^8^.

**Fig. 6:**
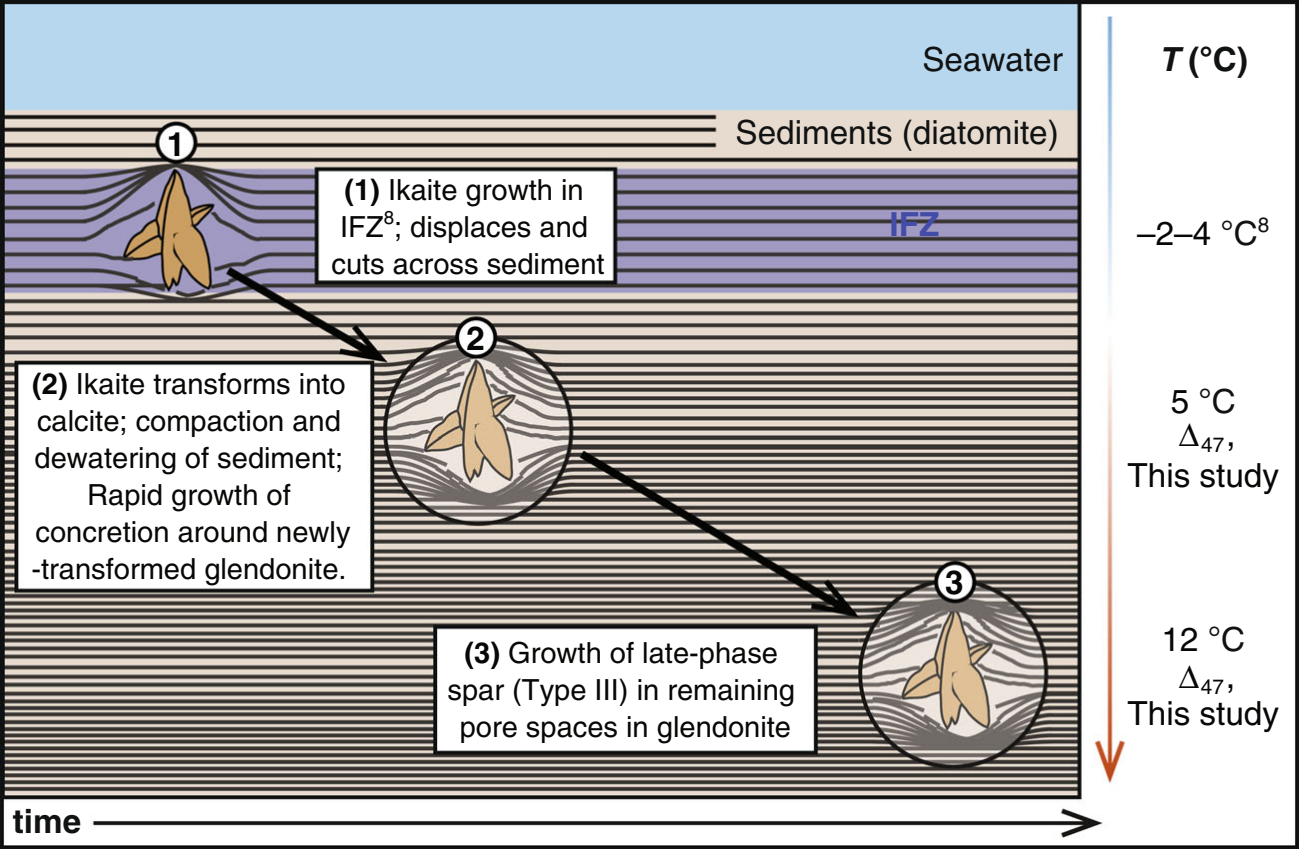
Model for ikaite growth and transformation to glendonite. This model is based on Zhou et al.^8^, Lu et al.^11^ and Vickers et al.^12^; clumped and stable isotopic and minor element analysis, and visual and microscopic observations on the Fur Formation glendonites (this study).

Burial diagenesis may alter clumped isotope compositions; however, burial heating of the Fur Formation was minimal (40–45 °C^46^, as further evidenced by the very high opal-A to opal-CT ratio^47,48^, very well-preserved diatoms and light-coloured and well-preserved dinoflagellate cysts, pollen and spores with very low colour alteration values (TAI 1 or TAI 1+; Dr. Claus Heilmann-Clausen, pers. comm. 2020; see Methods). Furthermore, such recrystallisation and reprecipitation reactions would bias samples to higher, rather than lower, Δ_47_ temperatures^49^. Similarly, higher Δ_47_ temperatures (relative to initial compositions) are expected for samples after partial reordering of their ^13^C-^18^O bonds^50^. However, it is currently not known what happens to ikaite Δ_47_ compositions during the transformation to calcite. It is possible that this transition results in disequilibrium Δ_47_ compositions that are colder than predicted. Such behaviour has been observed during controlled laboratory heating experiments when aragonite transforms to calcite^51^. In this case, we can rule out a clumped isotope effect during the ikaite-calcite transition because the Δ_47_ values of the primary (type I) calcite are indistinguishable from the concretionary calcite (type II), which did not experience such a transition. This lack of any significant statistical difference in Δ_47_ temperature between the concretions and the glendonites of the Fur Formation argue for a common early diagenetic phase of calcite precipitation when pore-water in the sediment was still close to the same temperature as the bottom-waters (Fig. 6). This is confirmed by our quality check using sub-samples of different calcites from the same sample from the Kola Peninsula, whereby the glendonite and sedimentary carbonate gave the same clumped isotope temperatures (within error) as mussel and bivalve shell aragonite from the same sample (see Methods and Supplementary Figs. 3–5). Consequently, our glendonite Δ_47_ temperatures may be taken as an upper limit for bottom water temperatures. The ikaite may have transformed into calcite at any point after it grew in the ikaite formation zone (Fig. 6). Mean Δ_47_ temperatures from the concretionary calcite are considerably colder than those for the Type III calcite (5 °C ± 4 °C vs 12 ± 5 °C), indicating that this spar grew after the concretion formed around the newly-transformed glendonite, at deeper burial depths (Fig. 6).

The reconstructed TEX_86_ SSTs from concretions and glendonites represent both a slightly older and longer duration time slice than the clumped isotope bottom water temperatures recorded in the glendonites. The sediments from which the biomarkers are derived were deposited, and the ikaite grew rapidly when they passed into the Ikaite Formation Zone, several metres below the sediment-water interface^8^, displacing and cutting across sedimentary laminae. Later, before significant compaction, the ikaite transformed into glendonite (type 1 calcite, Fig. 6). The concretion grew around the glendonite, yet across many laminae (Fig. 6) and so the extracted GDGTs from the concretions represent longer duration time slices than the biomarkers in the glendonite^34,35^, and both represent a longer time slice than the clumped isotope temperatures recorded in the Type 1 calcite (Fig. 6).

**Fig. 7:**
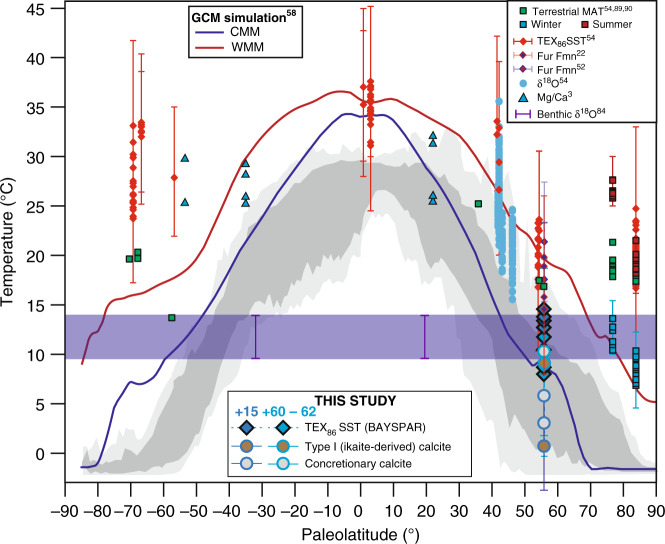
Compilation of early Eocene global temperature estimates. This spans the interval between PETM and ETM2/EECO (55 – 54 Ma^85^). New Δ_47_ bottom water temperatures and biomarker-derived terrestrial mean annual temperatures (MBT’/CBT, using the calibration of Peterse et al.^88^), foraminiferal δ^18^O temperatures, and TEX_86_ SSTs (recalibrated using BAYSPAR^31^ as compiled by Hollis et al.^54^. TEX_86_ SSTs from Stokke et al.^22^ show data from between ash horizons −13 and +101, excluding those with BIT > 0.4 and ΔRI > ± 0.3. Summer and winter terrestrial temperatures by bioclimatic analysis from Lomonosov Ridge^89^, and from Russia^90^. Reevaluated Mg/Ca data from the compilation of Evans et al.^3^. Error bars for biomarker and clumped isotope temperature reconstructions (both published and this study) denote the 95% confidence level. GCM simulations for EECO from Davies et al.^59^ show modelled mean SSTs for warm and cold months (red and blue lines, respectively), from the HadCM3L model, at 1200 ppm CO_2_. Modern mean annual temperature and seasonal range in SST^3^ are shown (dark- and light-grey shading, respectively). Bottom water temperature ranges from benthic foraminifera shown in blue shading^55^. CMM cold month mean, WMM warm month mean.

Recently published TEX_86_ SSTs from the upper Ølst and throughout the Fur Formation^22^ (from below ash horizon −39 and up to +101; Fig. 2) indicate that this cooling trend began at the top of the PETM interval (ash horizon −21a) and persisted throughout the entire interval, with temperatures reaching as low as 14.6 °C and averaging 17.3 °C. The SSTs for the +15 horizon from this study are even lower (14.2 °C in the +15 horizon and 10.4 °C in the +60–62 horizon); suggesting that the low resolution of the Stokke et al.^22^ study missed the coolest intervals. An earlier study^52^ reconstructed temperatures for the −17 horizon (c. 10 m lower stratigraphically than the +15 horizon, Fig. 2) as c. 18 °C, in close agreement to that for the same level from Stokke et al.^22^ (Fig. 7).

Whilst global temperatures show a general cooling after the PETM and before ETM2^2,53^ (Fig. 1), these SSTs and bottom water temperatures from the Danish Basin are considerably cooler than any other reconstructed annual SSTs or bottom water temperatures during this interval^54,55^ (e.g. Figs. 1c and 7). Indeed, the Polar Regions^54^ give average annual SSTs for the Arctic of 17–25 °C^56^, and c. 24–34 °C for the Antarctic^57,58^ (Fig. 7), precluding the possibility of annual freezing polar temperatures^2,59^. Thus, the very low surface and deep temperatures observed in the Danish Basin (and possibly the broader Nordic Seas region^4^) during the period between PETM and ETM2, appear to be a regionalised phenomenon. Stokke et al.^22^ propose that such cooling in the Nordic Seas region could be driven by multiple highly explosive eruptive phases of the nearby NAIP, which may have released sulphuric acid aerosols into the troposphere, where the sulphur has a residence time of weeks and could cause cooling on a regional, rather than global, scale^22^. Evidence for this hypothesis comes from the numerous, thick ash horizons in the post-PETM strata of the Fur Formation, and indeed glendonites are often found just below thicker ash horizons (Fig. 2). Furthermore, the unique paleogeography of the region might have promoted a bias towards winter temperatures in the bottom waters of the basin, leading to the conditions favourable for ikaite growth in the basin floor. Today, relatively cool and dense bottom shelf and slope waters form around many mid latitude continental shelf areas, by dense water cascades formed by evaporation and winter cooling or freezing^60^. Semi-enclosed or enclosed basins are particularly susceptible to such surface density increases, as wind-driven winter cooling of the surface can result in strong density differences between stratified surface and deep water layers^60,61^. For example, during cold, dry winters, in the Gulf of Lion and the Adriatic Sea, cold northerly Mistral and north-easterly Bora winds, respectively, bring cold dry air from the continent and cause such cascading events by surface cooling^61,62^. Such an effect is likely in the Eocene Danish Basin and/or Nordic seas considering the paleolatitude and paleogeography (Fig. 1); the prevailing wind direction in the Nordic seas is believed to be westerly with occasional cold winter winds from the North^63,64^. Such northerly cold winds could drive the surface cooling necessary to trigger cascading events that would bring cold surface waters down to the basin floor, where they flow southward, and, due to the stratification of the basin, gradually become de-oxygenated by the high productivity in the North Sea−Danish Basin. The relatively small scale of such cascading events, both in extent (0.5-1°) and duration (a few days), is testable in climate models, but at present below the scale of most current GCM simulations, which typically use a grid size of ~3°, and tend to generate monthly or yearly averages. Nonetheless, some GCM simulations show intermediate deep water formation in the proto-North Atlantic under some middle Eocene grid configurations^64^.

In summary, the temperatures derived by clumped isotope thermometry from the glendonites and concretions record a snapshot of low (c. 5 °C) bottom water temperatures during the Early Eocene that cannot be explained by diagenetic effects, and are consistent with current natural ikaite-bearing sites. Together with SSTs (representing a longer time span) of 8–15 °C derived from our TEX_86_ data, this study indicates that seawater temperature profiles in the Danish Basin are seemingly contrary to global temperature reconstructions for the earliest Eocene, on both short and long timescales. When coupled with observations of multiple glendonite-bearing horizons in Svalbard, and given a lack of similar findings in the Arctic during this time, it is evident that this observed cooling is a regional phenomenon. Multiple eruptive phases of the nearby North Atlantic Igneous Province have been proposed as the driver for such cooling, which resulted in much colder winters and triggered short-duration density-driven cascading events that brought cold water to the seafloor and promoted the growth of ikaite in the 45 °N Danish Basin. Whilst beyond the scope of this study, assessment of temperature changes across the glendonite-bearing horizons and into these thicker NAIP ash horizons, and high-resolution GCM simulations, are recommend for future studies to assess the magnitude and extent of such cold episodes.

## Methods

### Sample collection and preparation

The glendonites are found associated with carbonate concretions in the Fur Formation diatomite, close to or within ash horizons, in three intervals in the succession, namely, at or close to the +15 to +19 ash horizons, at the +51 horizon, and between the +60 to +62 layers (Fig. 2). They may be up to 1.5 m in diameter, and are found either within concretions or on their own. Glendonite and concretion samples were provided from the Museum Mors collection by Bo Pagh Schultz and Henrik Madsen. Samples of diatomite and volcanic ash were collected at the Ejerslev quarry, Mors.

The c. 0.5 m diameter concretions containing glendonites, and the individual glendonites not in concretions were cut in half vertically. The glendonite blades were also cut across a vertical and horizontal cross section, for thin section preparation).

### SEM, CL and light microscopy

Polished thin sections were examined using a polarising light microscope, and cathodoluminescence (CL) petrography was performed using a CITL Mk5-2 Optical Cathodoluminescence System, operating at an accelerating voltage of ≤10 kV and current of ≤265 μA. Selected thin sections were examined using a Quanta FEG 650 SEM at the University of Exeter Penryn Campus at 15 kV with a working distance of 12 mm using the backscatter electron detector. Element maps were collected using a double EDS detector; maps made using software provided by Bruker.

### Stable isotope and minor element analysis

Samples for stable isotope analysis were prepared by stepwise drilling horizontally across and vertically up the freshly cut surface of the concretion and glendonite (ca. 100 mm increments; ca. 2 mm drill depth; ca. 1 mm drill bit diameter) using a handheld drill (Supplementary Fig. 3). Minor element analyses were performed using an Agilent 5110 VDV ICP-OES at the Camborne School of Mines, University of Exeter. The minor element data are expressed as ratios to Ca. Carbon and oxygen isotope analysis of the calcite was performed at the University of Copenhagen using an Isoprime triple collector Isotope Ratio Mass Spectrometer using ~0.5 mg of powdered sample. Carbon and oxygen isotope ratios are expressed in standard delta notation as per mil deviation from the Vienna Pee Dee Belemnite (VPDB) standard.

The reproducibility of the measurements determined by the S.D. of in-house reference materials was 0.08% for δ^18^O and 0.04% for δ^13^C.

### Clumped isotopes

Brown (primary, ikaite-derived, Type I) calcite was separated from late-stage calcite spar (Type III calcite) by smashing portions of the glendonites and hand-picking the brown and transparent calcite into separate vials using tweezers. The separated brown and white calcite samples were then powdered using an agate mortar and pestle. Concretionary calcite samples were taken using a hand-held drill (1 mm drill bit).

To test whether glendonite calcite may be reliably used for clumped isotope thermometry, sub-samples of Recent carbonates from the Kola Peninsula were analysed (66**°**28′ N, 35**°**19′ E). Glendonite calcite (lacking the Type III spar), sedimentary carbonate, and mussel and bivalve aragonite from the same sample were analysed by clumped isotope thermometry following the same method as the Fur Formation carbonates (described below). The ∆_47_ values for Quality control test of Recent Kola Peninsula samples range between 0.748 and 0.761, yielding temperatures of 2–5 °C, all within error of each other at the 95 % ci (see Supplementary dataset).

Powdered samples were then transferred to ETH, Zurich for clumped isotope analysis. Clumped isotopes measurements were carried out at the ETH Zurich using a ThermoFisher Scientific MAT253 mass spectrometer coupled to a Kiel IV carbonate preparation device, following the methods described in Müller et al.^65^. The Kiel IV device included a PoraPakQ trap kept at −40 °C to eliminate potential organic contaminants. Samples were measured between November 2018 and July 2019 by measuring maximum 3 replicates of each sample per measuring session which consists generally of 24 samples of 130–150 µg interspersed with 20 replicates of each of the three carbonate standards ETH-1, ETH-2 and ETH-3^66^. The samples were analysed in LIDI mode with 400 s of integration of sample and reference gas. The calculations and corrections were done with the software Easotope^67^ using the revised “Brand parameters” for ^17^O correction as suggested by Daëron et al.^68^. The data are reported with respect to the carbon dioxide equilibration scale CDES. The Peirce elimination criterion was applied to detect and remove outliers^69^. This resulted in the elimination of two replicate analyses from the dataset out of a total of 98 measurements (see Supplementary dataset).

Temperatures were calculated using the Kele et al.^70^ calibration recalculated with the “Brand parameters” and the new accepted values for the ETH standards as reported in Bernasconi et al.^66^. The 1 S.D. uncertainties for the clumped isotope measurements, calculated from 86 replicate analyses are between 0.02‰ and 0.03‰. Potential isobaric interferences were monitored with the mass 49 parameter and the Δ_48_ offset as defined by John and Bowen^67^. The mean Δ_48_ offset value of the samples is 0.1 ± 0.3 (1 SD) ‰, and all replicates, with the exception of one measurement, have values lower than 0.7 ‰. The measurement with a higher Δ_48_ offset value (2 ‰) is from sample MLV gl 9; it was not removed from the dataset because it does not affect appreciably the sample mean (i.e., exclusion changes average by 1 ppm). The values obtained for the mass 49 parameter also do not suggest the presence of contamination. Samples have an average value of 0.02, and all replicates have values lower than 0.14.

### Biomarkers

Approximately 30 g of powdered, dry sample (glendonite, concretion, diatomite) was Soxhlet-extracted using DCM:MeOH 2:1. Sulphur was removed using activated copper turnings. Extracts were dried over Na_2_SO_4_ and aliquots of the total lipid extract were derivatised with BSTFA and pyridine for 1 h, dis-solved in ethylacetate, and analysed by GC-MS using a 30 m DB5 column of 0.25 mm ID and 0.25 um film thickness as previously described^35^. Positional diol isomers co-eluted, and were semi-quantified based on their characteristic *m/z* 271, 285, 299, 313, 327, 341, 355, 369^39^. A second aliquot was analysed for GDGTs (TEX_86_) and Alkenones (U^K^_37_’) using a Dionex LPG-U3400 (SDN) UHPLC liquid chromatography system and Thermo Scientific Q Exactive Focus mass spectrometer with Atmospheric Pressure Chemical Ionisation (APCI). The standard GDGT analysis method from Hopmans et al.^71^ was used, but slightly adapted to this high performance instrument employing a single Waters Acquity UPLC, BEH HILIC 1.7 µm (150 ×2.1 mm, 1.7 µm) column and precolumn at 40° C, a flow of 600 µl min −1 and starting at 5% B (isocratic from 0–3 min), rising to 18 % B at 5 min (isocratic 5–10 min), rising to 35% B at 15 min and 100% B at 17.4 min, with 2.6 min re-equilibration time, which slightly shortened the analysis time. APCI was carried out in positive polarisation mode, at a capillary temperature of 275 °C. Masses were scanned from *m/z* 200 to 2000, and resolved to 70,000 at *m/z* 200, with mass calibration carried out externally in electro spray ionisation mode, using auto-calibration Pierce LTQ Velos ESI positive ion calibration solution (n-butylamine, caffeine, MRFA, and Ultramark 1621). Alkenone isomers were separated by mass resolution at 10 ppm (using *m/z* 531.54994 (C37:2), 529.53429 (C37:3), 545.56559 (C38:2), and 543.54994 (C38:3)) and validity of UK’_37_ values was monitored using an alkenone standard (kindly provided by I. D. Bull, University of Bristol, and S. Rowland, University of Plymouth). GDGTs were identified based on retention times and accurate masses: using *m/z* 1302.32267 (GDGT-0), 1300.30702 (GDGT-1), 1298.29137 (GDGT-2), 1296.27572 (GDGT-3), 1292.24442 (Cren / Cren isomer); 1022.00967 (brGDGT-Ia), 1019.99402 (brGDGT-Ib), 1017.97837 (brGDGT-Ic), 1036.02532 (brGDGT-IIa+IIa’), 1034.00967 (brGDGT-IIb+IIb’), 1031.99402 (brGDGT-IIc+IIc’), 1050.04097 (brGDGT-IIIa+IIIa’), 1048.02532 (brGDGT-IIIb+IIIb’), and 1046.00967 (brGDGT-IIIc+IIIc’). Indices of sediments used during a Round Robin study^72^ (Supplementary Fig. 6A, B) were determined to compare our result with values from other laboratories. Sediment A gave a TEX_86_ value of 0.589 and a BIT value of 0.965 (round robin^72^ mean TEX_86_ 0.540 ± 0.053, BIT 0.954 ± 0.022), and sediment C gave a TEX_86_ value of 0.704 and a BIT value of 0.051 (round robin^72^ mean TEX_86_ 0.0702 ± 0.023, BIT 0.043 ± 0.013). Comparison with an in-house standard at the University of Bristol kindly provided by D. Naafs and R. Pancost gave a TEX_86_ value of 0.552 and a BIT value of 0.095 (TEX_86_ 0.55, BIT 0.07^73^; Supplementary Fig. 6C).

The Long Chain Diol Index (LDI) and fractional abundance of the C_32_ 1,15-diol were used for SST reconstructions, and as indicators for upwelling, nutrient or freshwater input^37,38,72,74^ (e.g. Diol index 2 and *F*C_32_; Table 2). U^K’^_37_ and the core-top calibration^37^ were used for SST calculations and BAYSPAR^31^ was used for conversion of TEX_86_ into paleotemperatures. The BIT value was calculated as defined by Hopmans et al.^75^ and was below 0.3 for all samples except for the sediments. However, the mass spectrometric method used is known to slightly overestimate BIT values^72^.

### Water depth reconstruction for the Eocene North Sea Basin

As discussed by Rasmussen et al.^76^ (p. 6) depth estimates for the Eocene sea of the mo-clay area (principally the islands of Mors and Fur) have ranged from 50 m^77,78^ to a few hundred metres^5^ (maximum 500 m) based on various fossil groups including diatoms and fishes. The Stolleklint Clay was clearly deposited at shallower conditions than the overlying Fur Formation^5^. Diatom assemblages of the Fur Formation are dominated by neritic taxa comprising e.g. *Stephanopyxis turri*, *Trinacria* spp. and *Paralia* spp.^79^, which also include frequent resting spores (e.g. *Pterotheca* spp. and *Chaetocero*s spp.). Strictly benthic taxa are rare (e.g. *Arachnodiscus* spp. cf.^79^) while so-called tychoplankton (benthic diatoms occasionally carried into the plankton) and planktic species are more common. Today, *Chaetoceros* resting spores are often common in coastal regions under influence of coastal upwelling^80^, which independently favour the upwelling hypothesis discussed above. The rare occurrence of strictly benthic diatoms together with the existence of different oceanic fishes challenge the hypothesis of a water depth at less than 50 m. Taken altogether, the fossil evidence suggests a neritic (shelf), low-energy paleoenvironment for the Fur Formation, possibly with an average depth fluctuating around 200 m.

### Burial heating reconstruction for the Fur Formation

The opal-A/-CT ratio may be recognised as an indicator of burial diagenesis because several studies have shown that the opal-A to opal-CT transformation is primarily time-temperature dependent^81^, but also pH variations of the pore fluids may affect the transformation^82^. Pedersen et al.^47^ calculated that the Fur Formation diatomite contains 45–65% opal, 30–45% clay minerals and 10% volcanic dust. The opal is by far dominated by biogenic opal-A (amorphous silica) from the diatom frustules with only traces of recrystallised opal-CT, leading to the assumption that dissolution of opal-A is at a minimum^47^. Petersen^48^ studied the mineralogy of scattered hardened beds in the lowermost 7 m of the Fur Formation and showed that the hardened beds are dominated by opal-CT interbedded with soft diatomite consisting primarily of opal-A. He speculated that the precipitation of opal-CT may have been related to the enormous volume of volcanic ash, which periodically was brought to the marine environment directly and from the surrounding terrestrial areas, where it subsequently was transported by rivers into the sea^48^. Amorphous silica (opal-A) dissolves and is replaced by opal-CT, usually at temperatures around 50–70 °C, which corresponds to about 1.5–2 km of overburden at average geothermal gradients^83^, but the depth of the opal-A/-CT transformation zone may vary considerably according to local physical, chemical and lithological conditions. In exploration well 35/10-1 in North Viking Graben in the Norwegian part of the North Sea, the opal-A/-CT transformation zone is situated at a burial depth close to 850 m^81^.

A very low degree of thermal alteration is supported by the excellent preservation of morphological characters in the diatom flora throughout the formation (Rasmussen, pers. obs.) and light-coloured and well-preserved dinoflagellate cysts, pollen and spores, which indicate very low colour alteration values (TAI 1 or TAI 1+) and only insignificant burial heating (Dr. Claus Heilmann-Clausen, pers. comm. 2020). Colour alteration is both temperature and time dependent, and it was estimated by Hartkopf-Fröder et al.^84^ that a TAI value of 2 from sediments heated for 10 Ma indicates a maximum burial temperature of almost 65 °C, while TAI 2 from sediments heated for 55 Ma indicates about 40–45 °C. Thus, TAI values of 1 or 1+ from the Fur Formation indicate immature conditions with maximum burial temperatures lower than these estimates. In addition, McNamara et al.^46^ (Supplementary Notes p. 6) estimated that the minimum burial depth of the Fur Formation was 100–150 m, corresponding to burial a temperature of 40–45 °C.

Thus, it may be estimated that the burial temperatures probably reached a level around 45 °C with quite similar minimum and maximum burial temperature estimates.

## Supplementary information

Supplementary Information

## Data Availability

Data generated during this study are available for download in a supplementary spreadsheet. Source data are provided with this paper.

## Source data

Source Data

## Code availability

No codes were generated during this study.
