# Supplementary material for: Cold spells in the Nordic Seas during the early Eocene Greenhouse
Source: Nat Commun. 2020 Sep 18;11:4713. doi: 10.1038/s41467-020-18558-7 (PMC7501286; doi:10.1038/s41467-020-18558-7)
Supplement: Supplementary file 1 — Supplementary Information [file 41467_2020_18558_MOESM1_ESM.pdf]

**Supplementary Figures**

for *Cold spells in the Nordic Seas during the early Eocene Greenhouse*

Vickers et al.

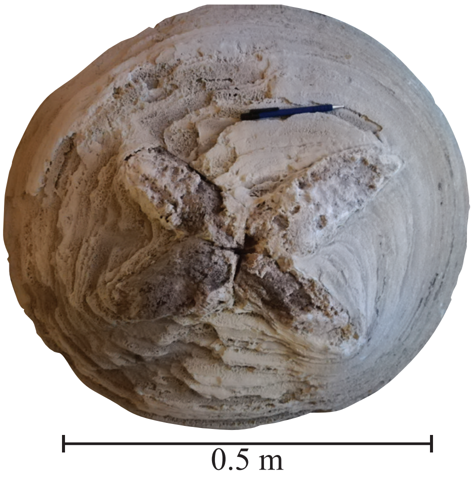


**Supplementary Figure 1: Glendonites of the Fur Formation.** Examples of glendonites from the +15 horizon (left) and +60 - +62 horizons (middle and right)

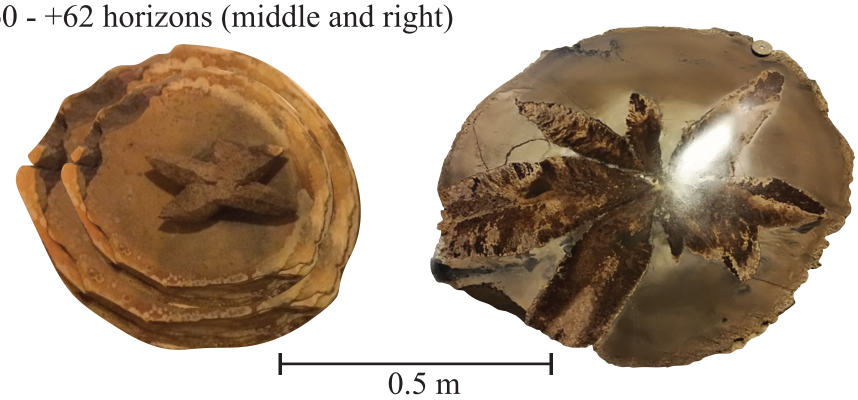


**Supplementary Figure 2: Sampled glendonites from the +15 horizon and +62 horizons.** Numbered dots indicated where samples were taken (See Supplementary dataset).

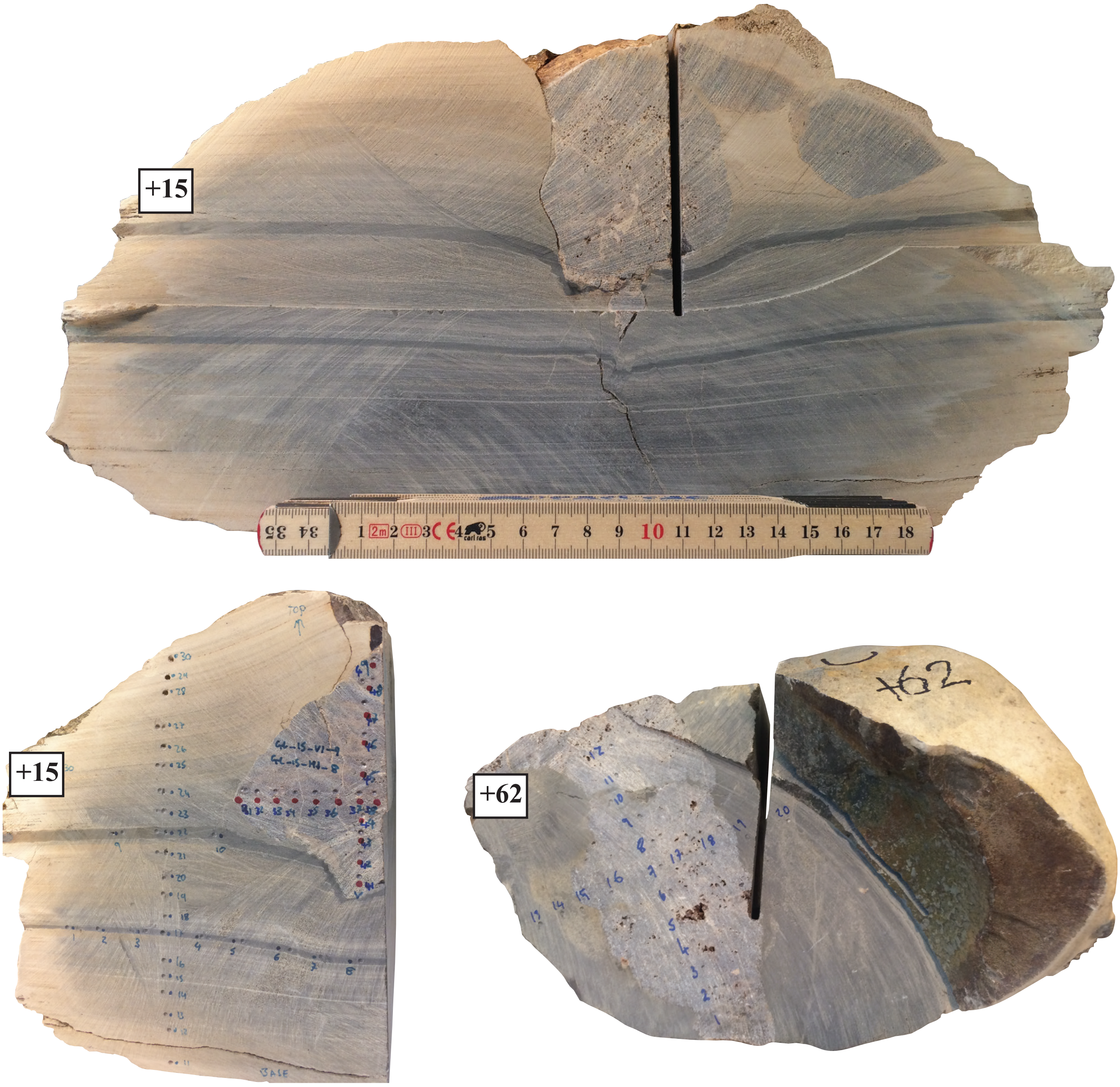

**Supplementary Figure 3: Recent carbonate samples for comparison to ancient glendonites.** Single sample containing Recent glendonite, mussels and bivalves in sedimentary carbonate from Olenitsa, Kola Peninsula. Labels show where analysed carbonates were taken.

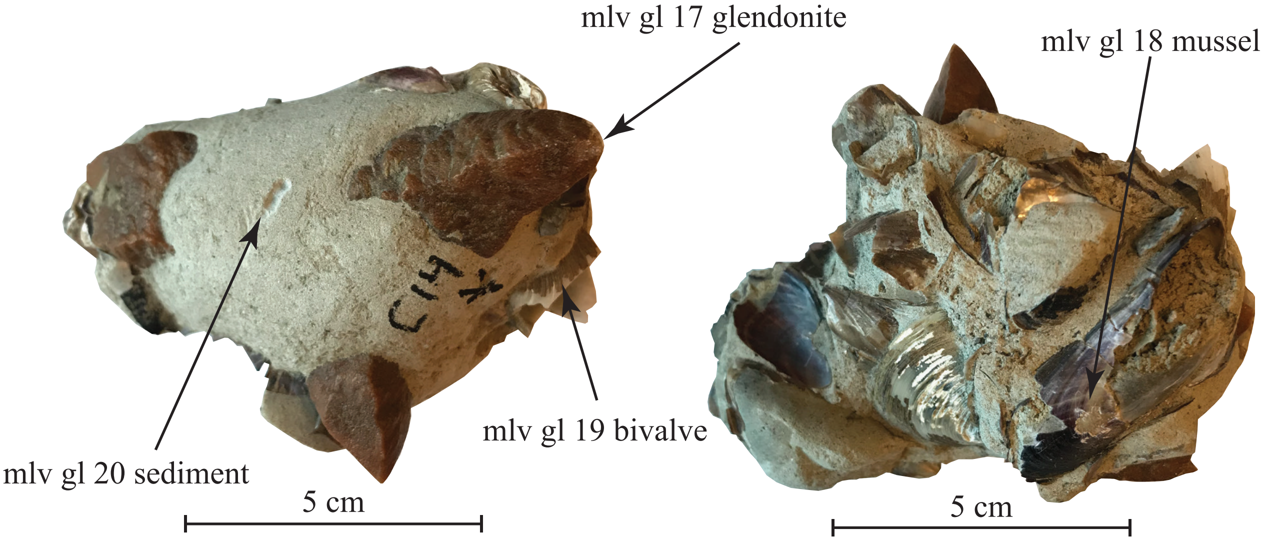


**Supplementary Figure 4: Stable isotopes of Kola Peninsula carbonates.** The early diagenetic glendonite and sedimentary carbonate are distinct from the biogenic carbonates, showing more negative $\delta^{13}C$ but more positive $\delta^{18}O$ than the bivalve and mussel.

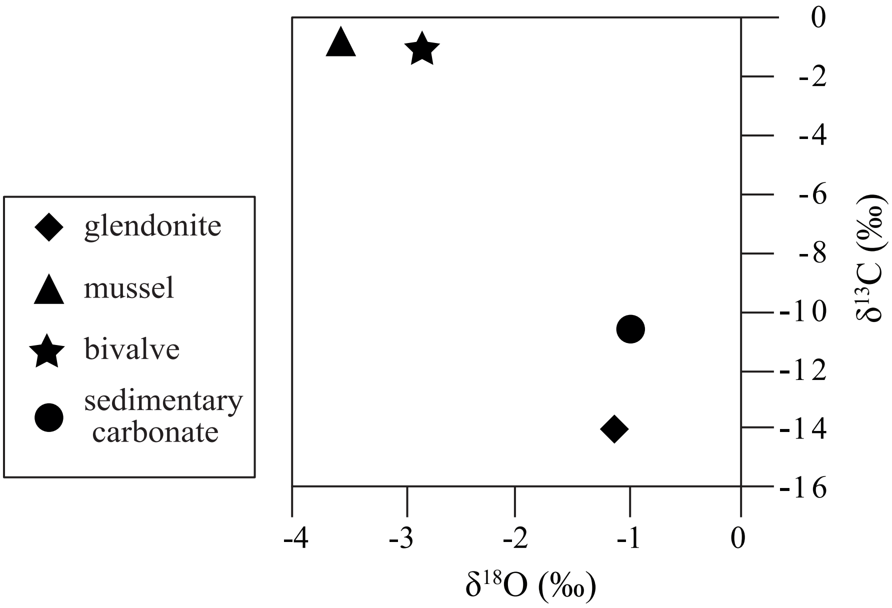

**Supplementary Figure 5: Comparison of Recent and Eocene glendonite samples.** Clumped isotope temperatures and reconstructed $\delta^{18}O_w$ (using the equation of Kim and O'Neil, 1997) for the Recent Kola Peninsula and Eocene Fur Formation carbonates. The 4 different carbonate types from the Kola Peninsula are within error of eachother, indicating that Type I glendonite calcite is represetnative of true bottom water temperatures.

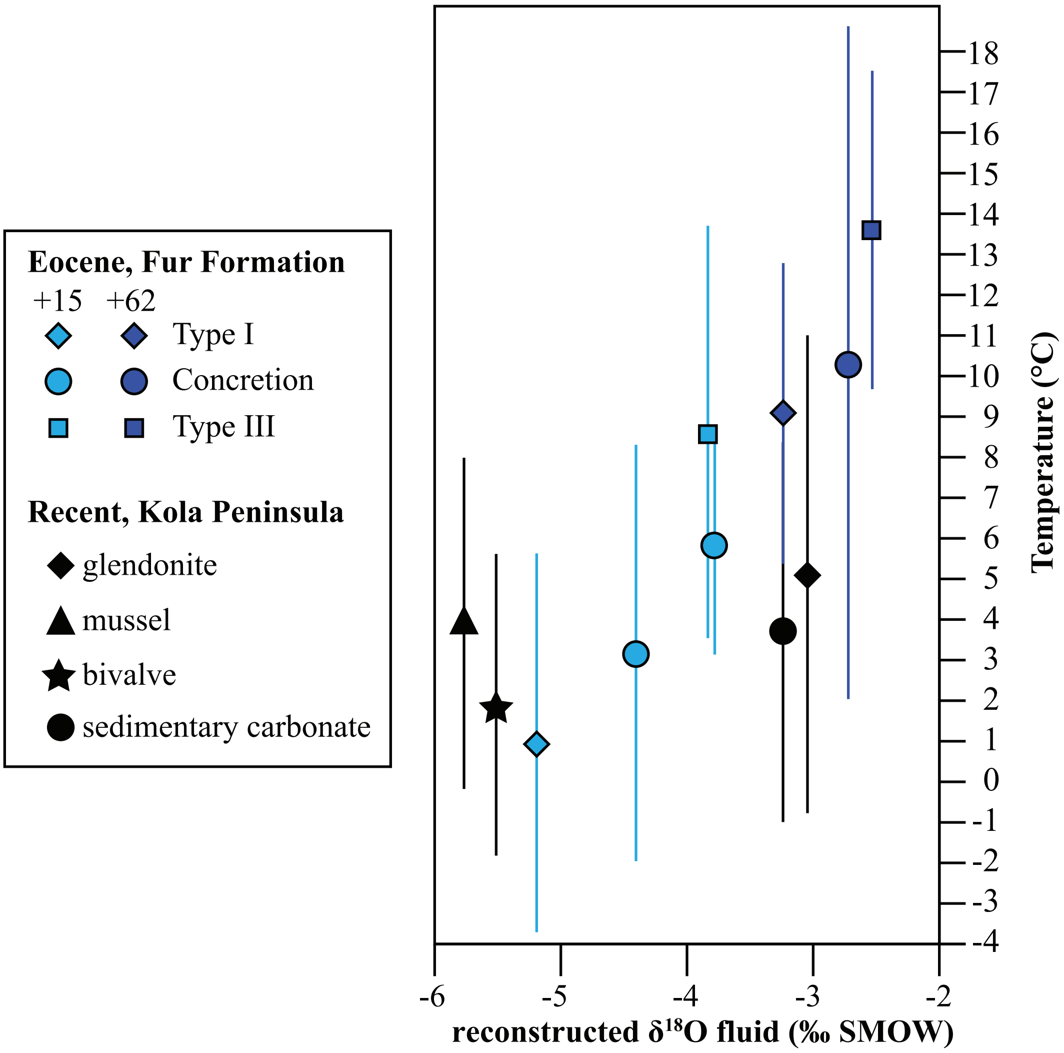

**Supplementary Figure 6: Chromatograms of biomarker extracts from sediments used for interlab comparison.** This shows the GDGTS used in the $TEX_{86}$ and BIT calculations, only combined traces for accurate masses of the GDGTs (see methods section), with 10 ppm mass accuracy.
**(A)** Sediment A[80]; **(B)** Sediment C[80]; **(C)** Marine Sediment[82].

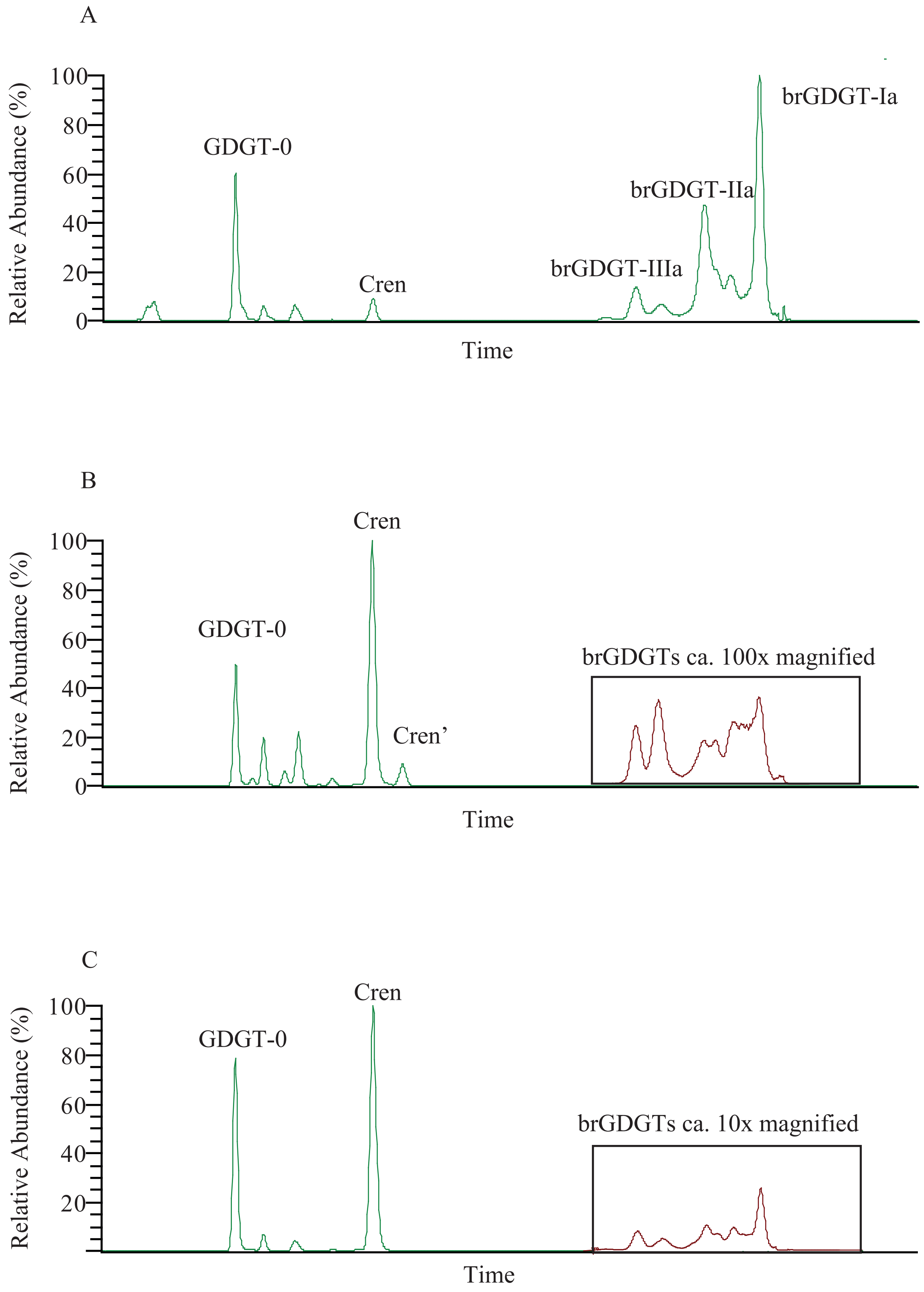

**Supplementary Figure 7: Chromatograms for the Fur Formation samples.** This shows the combined traces for accurate masses with 10 ppm mass accuracy, for Fur Formation glendonites, sediments and concretions fromt the +15 and +60 - 62 horizons.

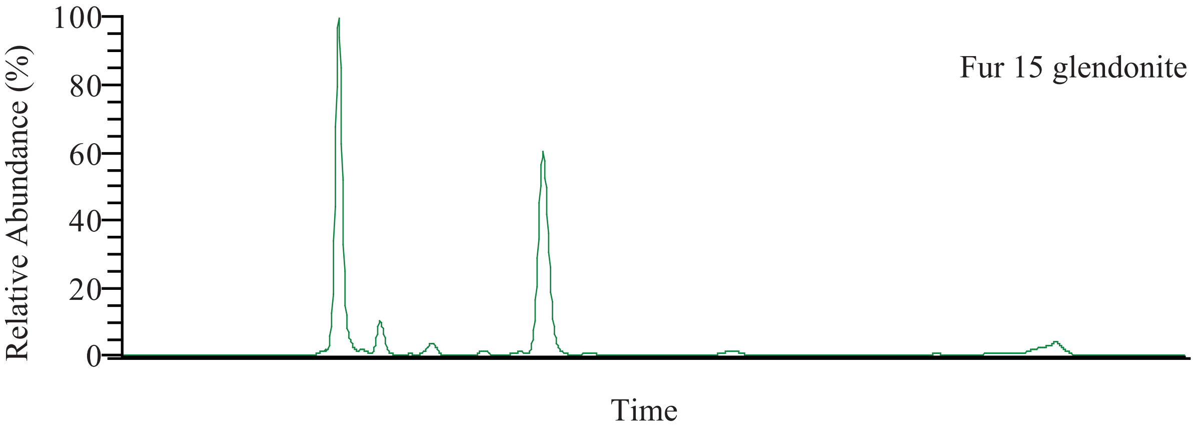


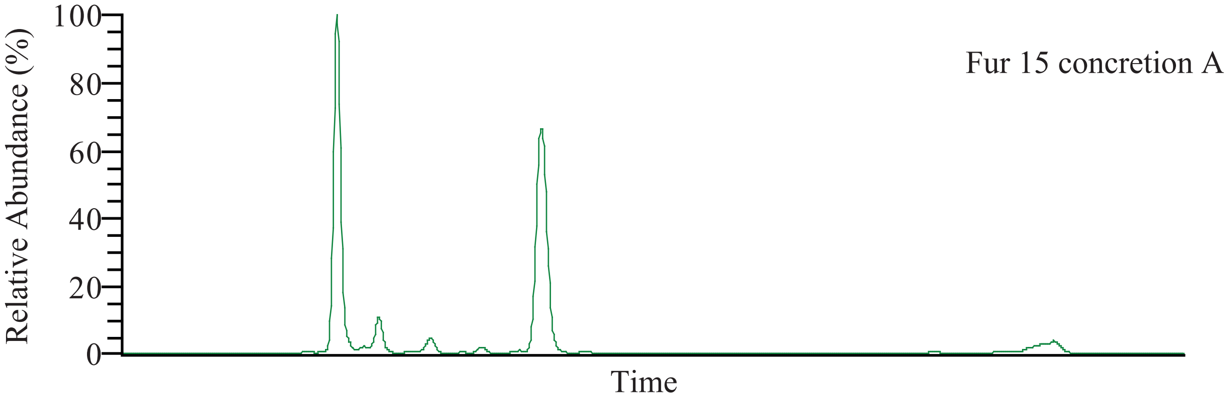


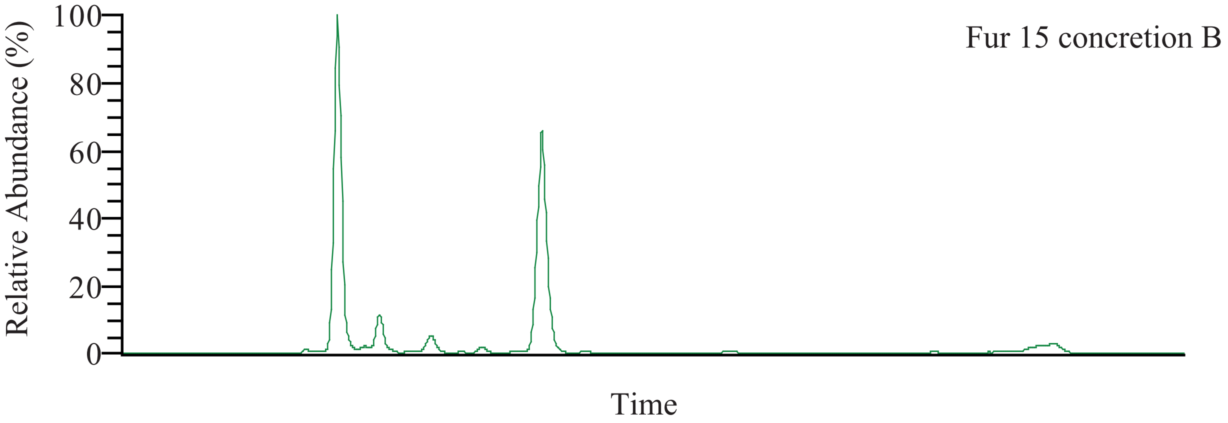

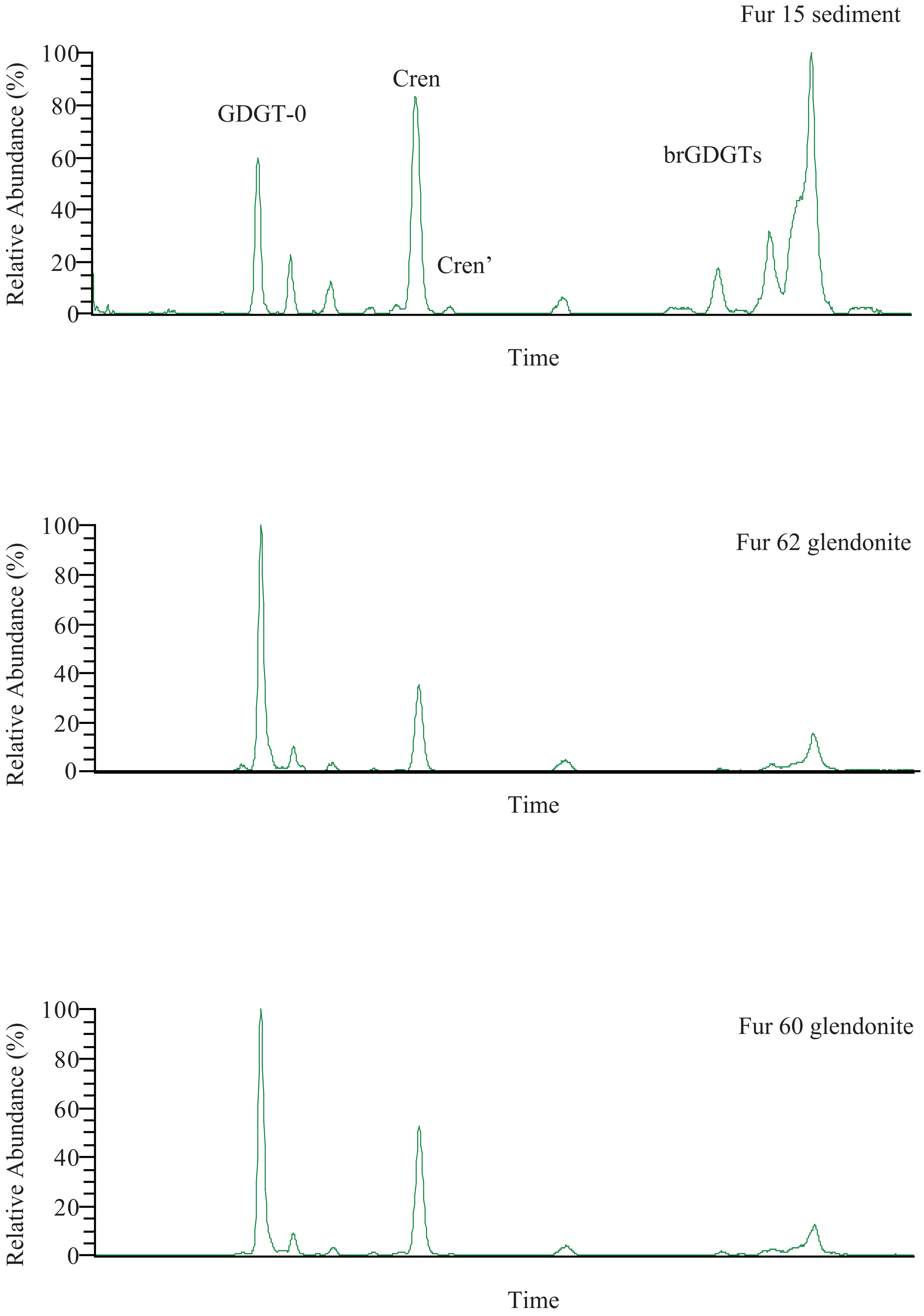

Fur 15 sediment
Relative Abundance (%)
100
80
60
40
20
0
GDGT-0
Cren
Cren'
brGDGTs
Time
Fur 62 glendonite
Relative Abundance (%)
100
80
60
40
20
0
Time
Fur 60 glendonite
Relative Abundance (%)
100
80
60
40
20
0
Time

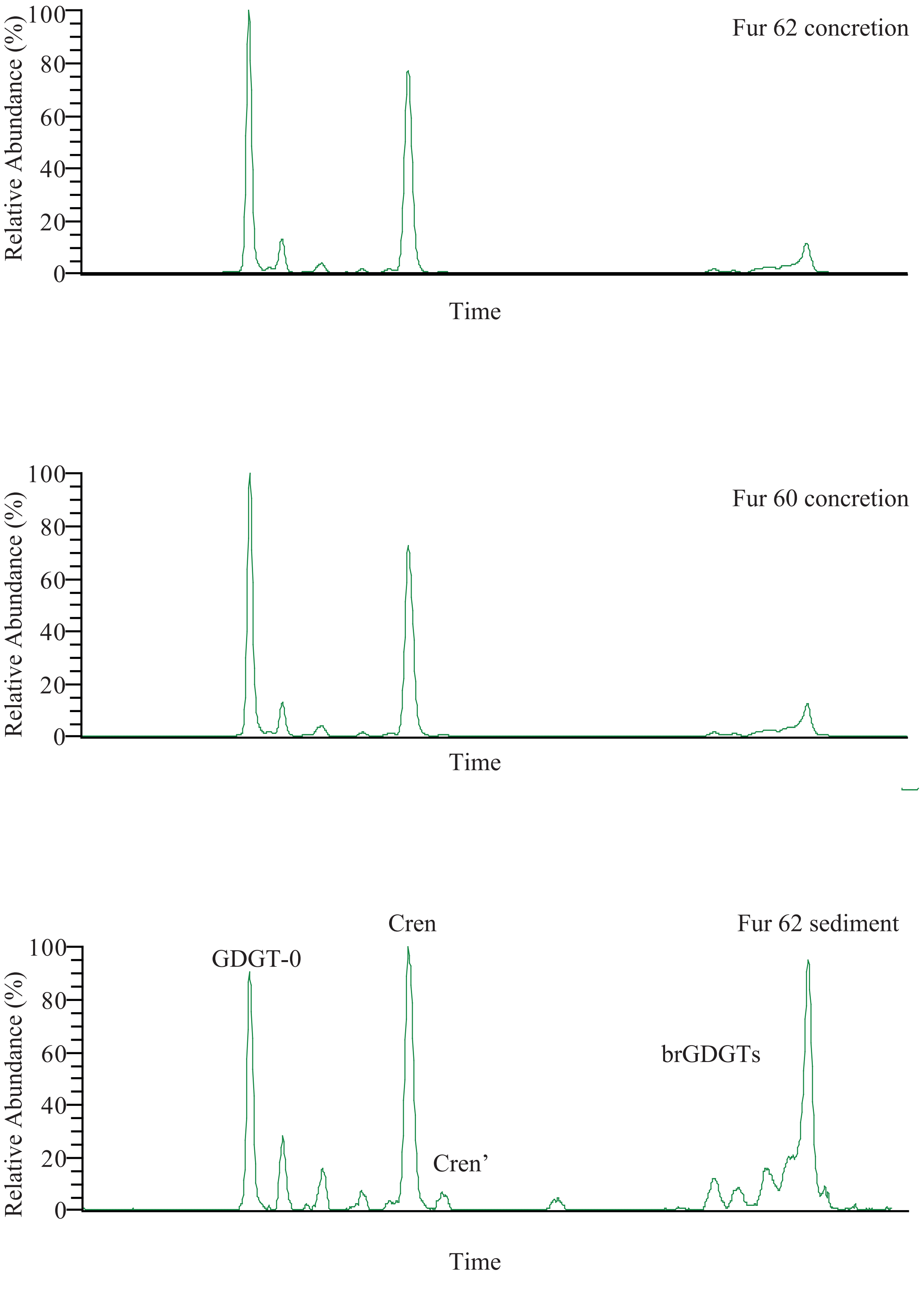
Fur 62 concretion
Relative Abundance (%)
100
80
60
40
20
0
Time
Fur 60 concretion
Relative Abundance (%)
100
80
60
40
20
0
Time
Fur 62 sediment
GDGT-0
Cren
Cren’
brGDGTs
Relative Abundance (%)
100
80
60
40
20
0
Time
